# Metal–dielectric hybrid nanoantennas for efficient frequency conversion at the anapole mode

**DOI:** 10.3762/bjnano.9.215

**Published:** 2018-08-27

**Authors:** Valerio F Gili, Lavinia Ghirardini, Davide Rocco, Giuseppe Marino, Ivan Favero, Iännis Roland, Giovanni Pellegrini, Lamberto Duò, Marco Finazzi, Luca Carletti, Andrea Locatelli, Aristide Lemaître, Dragomir Neshev, Costantino De Angelis, Giuseppe Leo, Michele Celebrano

**Affiliations:** 1 Matériaux et Phénomènes Quantiques, Université Paris Diderot - Sorbonne Paris Cité, CNRS UMR 7162, 10 rue A. Domon et L. Duquet, 75013 Paris, France; 2 Department of Physics, Politecnico di Milano, Piazza Leonardo Da Vinci 32, 20133 Milano, Italy; 3 Department of Information Engineering, University of Brescia, Via Branze 38, 25123 Brescia, Italy; 4 Centre de Nanosciences et de Nanotechnologies, CNRS-UMR9001, Route de Nozay, 91460 Marcoussis, France; 5 Nonlinear Physics Centre, Research School of Physics and Engineering, Australian National University, 2601 ACT Canberra, Australia

**Keywords:** nanophotonics, nonlinear optics, plasmonics, second-harmonic generation, semiconductors

## Abstract

**Background:** Dielectric nanoantennas have recently emerged as an alternative solution to plasmonics for nonlinear light manipulation at the nanoscale, thanks to the magnetic and electric resonances, the strong nonlinearities, and the low ohmic losses characterizing high refractive-index materials in the visible/near-infrared (NIR) region of the spectrum. In this frame, AlGaAs nanoantennas demonstrated to be extremely efficient sources of second harmonic radiation. In particular, the nonlinear polarization of an optical system pumped at the anapole mode can be potentially boosted, due to both the strong dip in the scattering spectrum and the near-field enhancement, which are characteristic of this mode. Plasmonic nanostructures, on the other hand, remain the most promising solution to achieve strong local field confinement, especially in the NIR, where metals such as gold display relatively low losses.

**Results:** We present a nonlinear hybrid antenna based on an AlGaAs nanopillar surrounded by a gold ring, which merges in a single platform the strong field confinement typically produced by plasmonic antennas with the high nonlinearity and low loss characteristics of dielectric nanoantennas. This platform allows enhancing the coupling of light to the nanopillar at coincidence with the anapole mode, hence boosting both second- and third-harmonic generation conversion efficiencies. More than one order of magnitude enhancement factors are measured for both processes with respect to the isolated structure.

**Conclusion:** The present results reveal the possibility to achieve tuneable metamixers and higher resolution in nonlinear sensing and spectroscopy, by means of improved both pump coupling and emission efficiency due to the excitation of the anapole mode enhanced by the plasmonic nanoantenna.

## Introduction

Second-harmonic generation (SHG) in bulk materials, first demonstrated by Franken and co-workers in 1961 [1], is nowadays successfully applied in a variety of disciplines. Besides its extended application in laser science for the realization of coherent light sources [2], SHG is a fundamental tool for the analysis and characterization of the crystal structure of solid materials [3–5]. The background-free character of SHG makes it an attractive tool for imaging biological tissues [6] and investigating structural and conformational properties of molecules at liquid–liquid interfaces [7]. The ability to downscale nonlinear optical processes, such as SHG, in extremely confined spatial regions opens many fascinating opportunities in light manipulation and multiplexing [8] as well as in optical sensing and spectroscopy [9–10]. Yet, to date, realizing nonlinear optical processes at the nanoscale remains a challenging task since phase-matching cannot be exploited as enhancement mechanism in systems confined below the wavelength of light. Exploiting the intense field enhancements stemming from plasmonic resonances in metallic nanoantennas is instead one of the most successful approaches to compensate for the lack of phase-matching and long interaction lengths at the nanoscale [11]. However, metals display large material losses in the visible range and limit the penetration depth of electric fields, thereby hindering the beneficial effects induced by the field enhancements. Moreover, centrosymmetry in metals results in extremely small efficiencies for second-order processes, a limitation that can be partly circumvented by designing individual nano-antennas [12] and extended array arrangements [13–14] featuring a lower degree of symmetry. Metal-less nanophotonics based on dielectrics of high refractive index and semiconductors recently emerged as a promising alternative to plasmonic nanostructures for linear and nonlinear nanophotonic applications due to the reduced losses at optical frequencies [15]. Since in high-index dielectric materials the electric field penetrates deeply into the volume [16], the exploitation of large bulk nonlinearities also enables enhanced nonlinear light–matter interactions at the nanoscale. Third-harmonic generation (THG) was the first nonlinear effect observed in nanoscale semiconductors with sizeable efficiency enhancement. First reported in individual silicon-on-insulator nanodisks [17] and soon after in a coupled nanodisk trimer configuration [18], the THG enhancement attained was up to 100 times higher than in a Si slab of the same thickness thanks to the exploitation of Mie-type resonances. Even higher THG efficiency enhancement has been recently achieved in germanium nanodisks thanks to the excitation of the so-called anapole mode (from the ancient Greek “without any pole”) [19]. As the name suggests, the anapole mode consists in the superposition of a toroidal dipole (TD) and an electric dipole (ED) mode with a π-phase difference, which results in transparency at the anapole wavelength and a high energy stored inside the material [20–21]. The extremely confined fields in the resonator along with the relative weak coupling of the resonator to the external radiation allow for a boost of the quality factors in these Mie resonators. This peculiar feature holds great potential to further enhance light–matter interaction. For this reason, these systems are currently the subject of intense investigations and strategies to attain light absorption enhancement [21], nonlinear amplification [22] and enhanced Raman scattering [23] have been recently suggested.

In this framework, Al*_x_*Ga_1−_*_x_*As, a III–V semiconductor, has become a popular material for nonlinear photonics thanks to its non-centrosymmetric structure and other important key assets including: i) a large band gap enabling TPA-free operation at 1.55 μm, ii) a high non-resonant quadratic susceptibility (*d*_14_ ≈ 100 pm/V for GaAs in the near infrared), and iii) a broad spectral window of transparency in the mid-infrared (up to 17 μm), which allows for the generation of intense second-order nonlinear optical effects. Many results have already been achieved in integrated nonlinear optics [24], including SHG in quasi-phase-matched waveguides [25], efficient frequency-comb generation [25], and optical parametric oscillation [26]. Only very recently, thanks to the dramatic improvement of nanofabrication techniques, the integration of semiconducting materials has been pushed even further with the realization of nanoscale platforms featuring efficient second-order nonlinear processes. Recently, a nanoscale system based on AlGaAs nanodisks pumped in the telecom range (λ ≈ 1554 nm), at coincidence with the magnetic dipolar resonance, was theoretically proposed as an efficient system to enhance second-order nonlinear effects in nanoscale optics [27]. Soon after, three independent experiments validated these theoretical predictions [28–30]. The conversion efficiencies reported were higher than 10^−5^, which is more than four orders of magnitude higher than in optimized plasmonic nanoantennas pumped at similar intensities [12]. Concurrently, promising studies on enhanced SHG at the nanoscale have also been performed on perovskite nanoparticles [31–32].

Several strategies that involve coupled nanosystems have been employed to enhance both second and third harmonic nonlinearities. For example, we recently reported enhanced SHG by closely coupling two AlGaAs nanopillars in a dimer configuration and by tuning the pump fundamental frequency to the anapole mode [33]. A further viable strategy involving coupled nanosystems, consists in placing a metal nanostructure in the proximity of the dielectric nanoantenna to manipulate the in- and out-coupling of light [34–35]. This hybrid integration was exploited to significantly boost the nonlinear conversion efficiency of nanosystems [32,36–37]. In particular, Maier and co-workers proposed a nanoantenna composed of a silicon disk core surrounded by an annular plasmonic antenna, which combines the energy-storage capabilities of the anapole mode with the enhanced efficiency of light-coupling in metal–dielectric systems. This allowed them to achieve a THG efficiency enhancement up to three orders of magnitude with respect to the bare disk [37]. In this work, we apply the same approach to obtain SHG enhancement in AlGaAs nanostructures [27–28,33] and investigate the properties of the emitted nonlinear optical signal. We find that the SHG yield at the anapole mode in these hybrid platforms is almost two orders of magnitude higher than in isolated nanopillars. We also find sizeable THG with an emission yield comparable to that of SHG, which is unexpected since third-order nonlinear processes are commonly negligible in these systems [28,38]. The polarization-dependent optical properties of this platform together with the SHG angular emission characteristics indicate an improvement in both pump coupling and emission efficiency. We hence obtain solid indications for the realization of a new class of nano-photonic platforms for nonlinear light manipulation at the nanoscale.

## Results and Discussion

**Figure 1:**
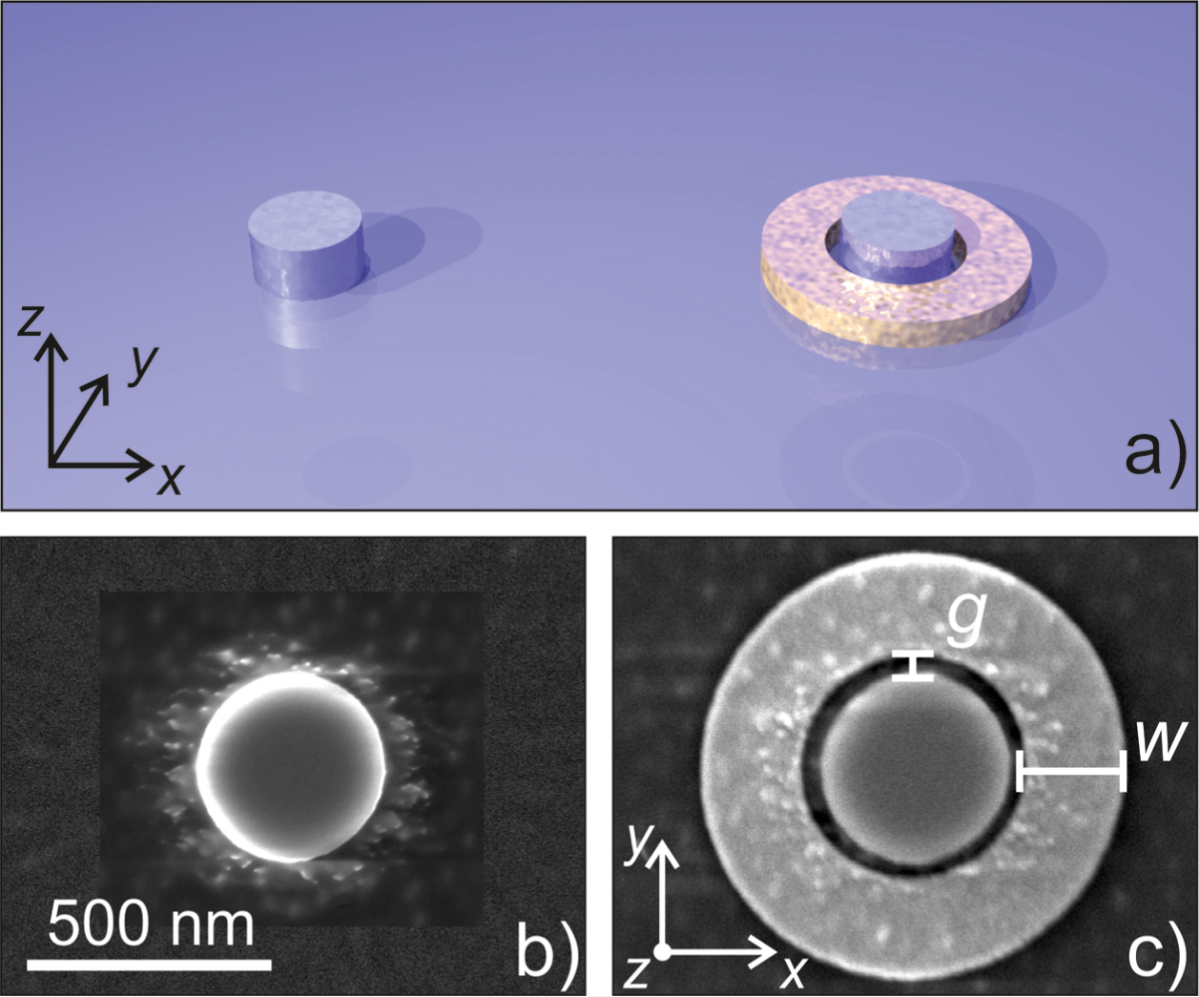
(a) Schematic of the investigated sample: plain AlGaAs nanopillar serving as a reference structure (left) and a hybrid structure consisting of a nanopillar with the same geometrical parameters as the reference but surrounded by an Au ring (right). (b) SEM image of a reference nanopillar (*r*_1_ = 380 nm). (c) SEM image of a hybrid structure (*r* = 410 nm, *h* = 200 nm, *g* = 100 nm, *w* = 410 nm).

Our plasmonic-dielectric hybrid nanostructures consist of AlGaAs nanodisks surrounded by an Au ring (Figure 1). Individual nanodisks (Figure 1b) and Au rings with the same geometrical parameters are also fabricated as a reference. We consider two distinct geometries: type 1 features a nanopillar with nominal radius *r*_1_ = 410 nm, height *h* = 200 nm and gap size *g*_1_ ≈ 100 nm as shown in Figure 1c, while type 2 features a nanopillar radius *r*_2_ = 380 nm with the same height and a gap size *g*_2_ = 200 nm (not shown). The geometrical parameters of the Au ring in both cases are *w* = 410 nm and *h* = 80 nm. At the fundamental wavelength the plasmonic ring produces a strong electric field at its center. This allows for an improved coupling to the toroidal dipole moment inside the disk, therefore providing a more efficient excitation of the anapole mode (i.e., ED and TD moments of equal magnitude and in phase opposition) at the fundamental wavelength (λ = 1554 nm).

**Figure 2:**
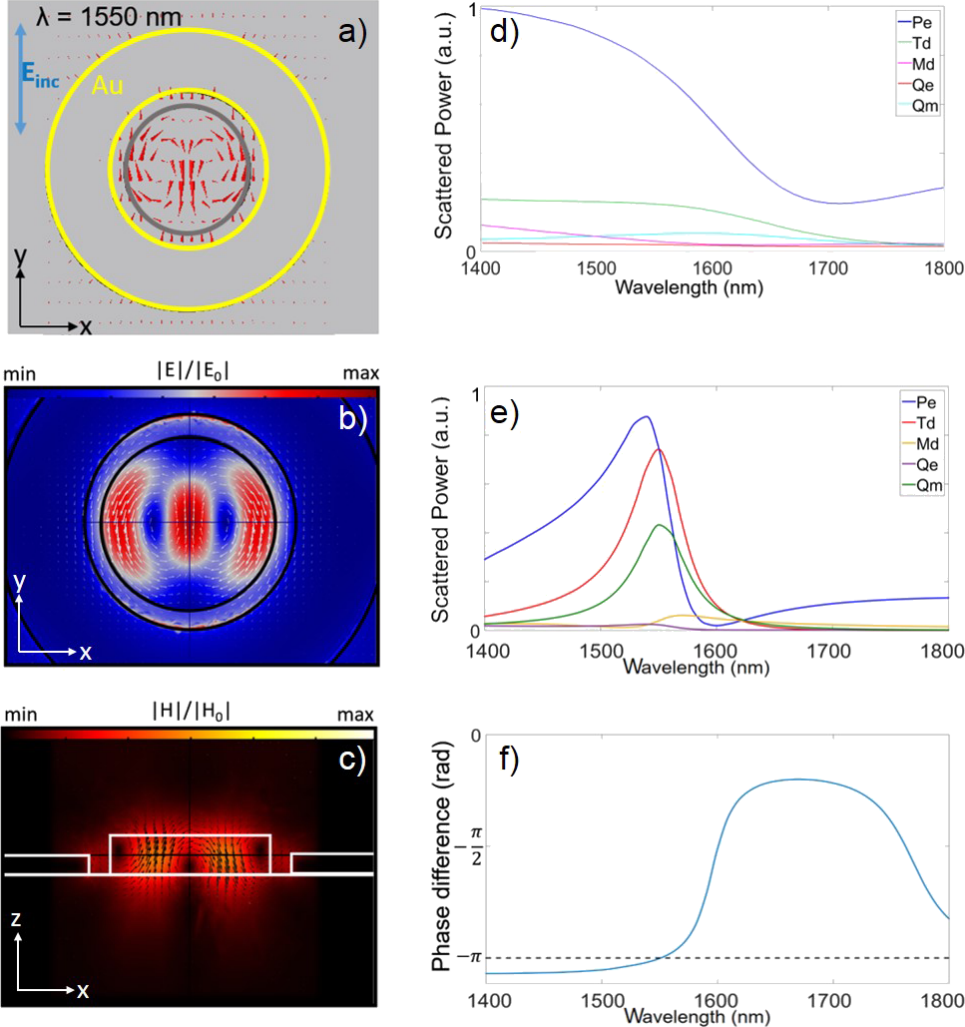
a) Electric field vector map at 1550 nm for the proposed structure. b) Electric and c) magnetic field distribution in the hybrid nanoantenna at 1550 nm. d) The full multipolar decomposition of the first five contributing multipole moments: electric dipole (Pe), magnetic dipole (Md), toroidal dipole (Td), electric quadrupole (Qe), magnetic quadrupole (Qm) for and isolated pillar and e) in the hybrid structure. f) Phase difference between Cartesian dipole moment Pe and toroidal moment Td of the induced current inside the cylinder in the hybrid structure.

We investigated the scattering characteristics of this structure at near-IR wavelengths by using finite element method (FEM) simulations in COMSOL. The incident light is a plane wave with a wave vector, **k**, parallel to the cylinder axis and the electric field, **E**_0_, polarized along the *y*-axis with respect to the reference system of Figure 2a. For the dispersion of the refractive index of Al_0.18_Ga_0.82_As we used the analytical model proposed in [39]. In Figure 2b,c both the electric and magnetic field enhancements are depicted, respectively, which help identifying the typical anapole configuration. We have compared the linear behavior of the hybrid geometry against that of a bare nanodisk with its anapole mode at correspondence with the pump wavelength. To do so we focused on two key parameters: the total scattered power, calculated as the surface integral of the Poynting vector normal to an imaginary sphere enclosing the entire structure, and the internal energy defined as the volume integral of the squared electric field inside the cylinder.

**Figure 3:**
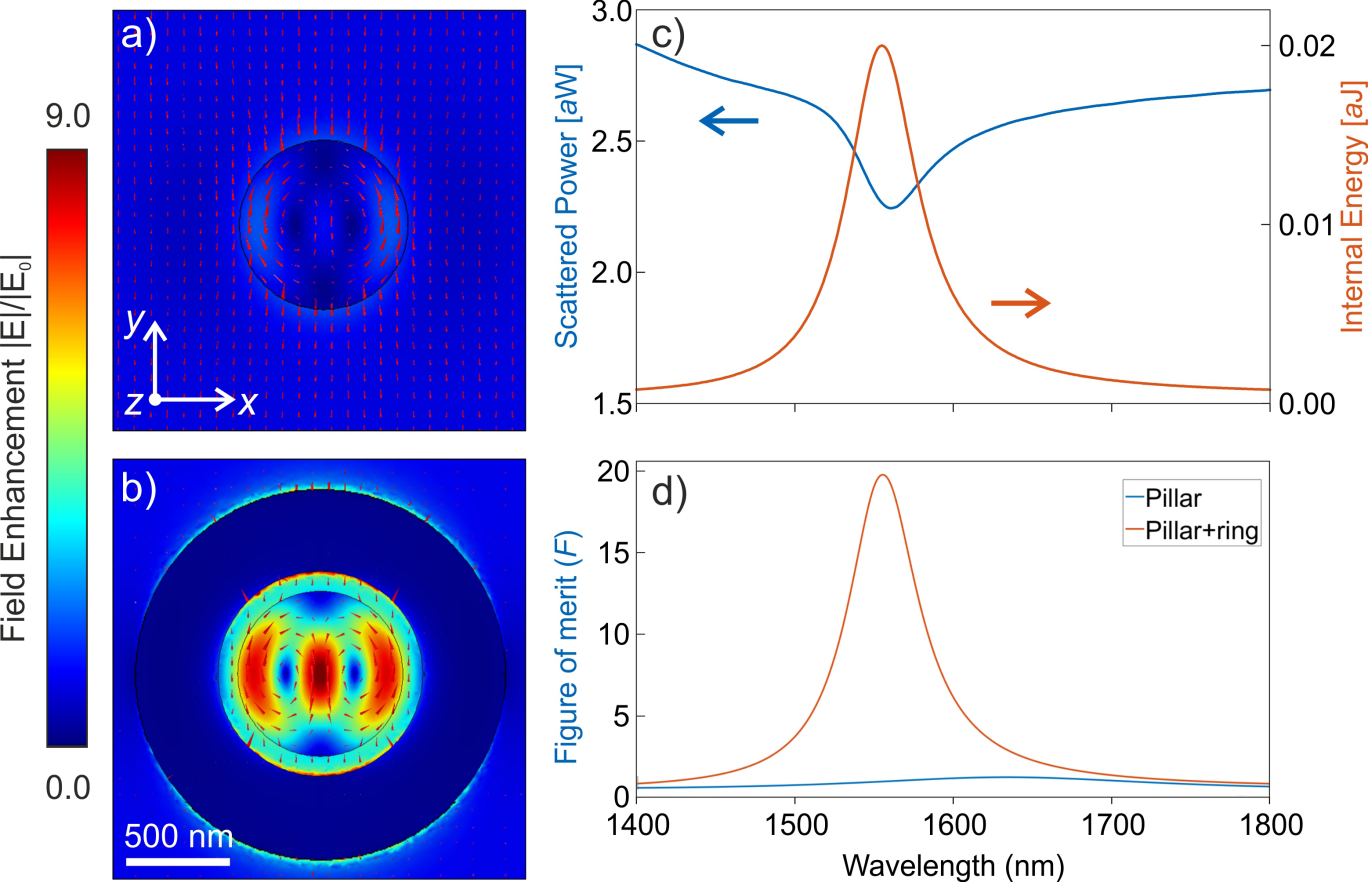
a) Electric field enhancement at 1550 nm in the *xy*-plane for the isolated type 1 (*r* = 410 nm) nanocylinder and b) for its respective hybrid structure. c) Total scattered power (blue curve) and internal energy stored (orange curve) by the anapole in the pillar as a function of the wavelength. d) Normalized electric field intensity averaged within the cylinder, *F*, as a function of pump wavelength for the isolated AlGaAs cylinder (blue curve) and for the hybrid nanoantenna (orange curve).

To gain further insights into this behavior, in Figure 2d (2e) we show the contributions to the scattered power of the first five radiating multipole moments in the bare nanodisk (hybrid system) we computed the first five Cartesian multipole moments inside the cylinder using the expressions summarized in [40], where Pe, Md, Td, Qe and Qm indicate the electric dipole, magnetic dipole, toroidal dipole, electric quadrupole and magnetic quadrupole moments, respectively. The multipole decomposition reveals that, while the electric dipole is dominant in the bare nanodisk, both the electric and toroidal dipole components are dominant inside the cylinder when the gold ring is present. In particular, they have the same magnitude and a phase difference close to −π (see Figure 2f) for λ = 1550 nm. Let us recall that, to get the exact anapole condition, the two abovementioned dipoles must be in phase opposition and exhibit the same magnitude while all the other contributions are zero. Unlike the ideal case, in our situation, there is a magnetic quadrupole contribution that slightly perturbs the anapole condition. Looking at the electric field inside the cylinder, it is possible to observe the typical anapole field distribution (see upper panel of Figure 2a) where almost the whole E-field amplitude circulates in the plane of the disk and is confined inside it. We also evaluated the electric field enhancement inside the cylinder, defined as the ratio between the total field *E* and the incident field *E*_0_, as a figure of merit to assess the performance of the ring-assisted antenna. The maximum field enhancement is about six times higher in the hybrid structure than in the isolated cylinder, as shown by the comparison between Figure 3a and Figure 3b. Figure 3c displays the wavelength-dependent total scattered power and internal energy stored in the structure, which confirm the anapole-like behavior at the pump wavelength. By engineering the geometrical parameters of dielectric nanoresonators working at the anapole mode, Wang and Dal Negro [21] demonstrated that light absorption can be enhanced. While this approach proved effective to finely tune the anapole condition, the pump light absorption and, therefore, the field enhancement inside the structure remains rather limited. In this work, by exploiting the plasmonic ring, we obtain a 20-fold increase in the field intensity enhancement averaged over the whole structure volume, *F*, as compared to the bare nanopillar (see Figure 3d), which is comparable to what was already reported in [37].

**Figure 4:**
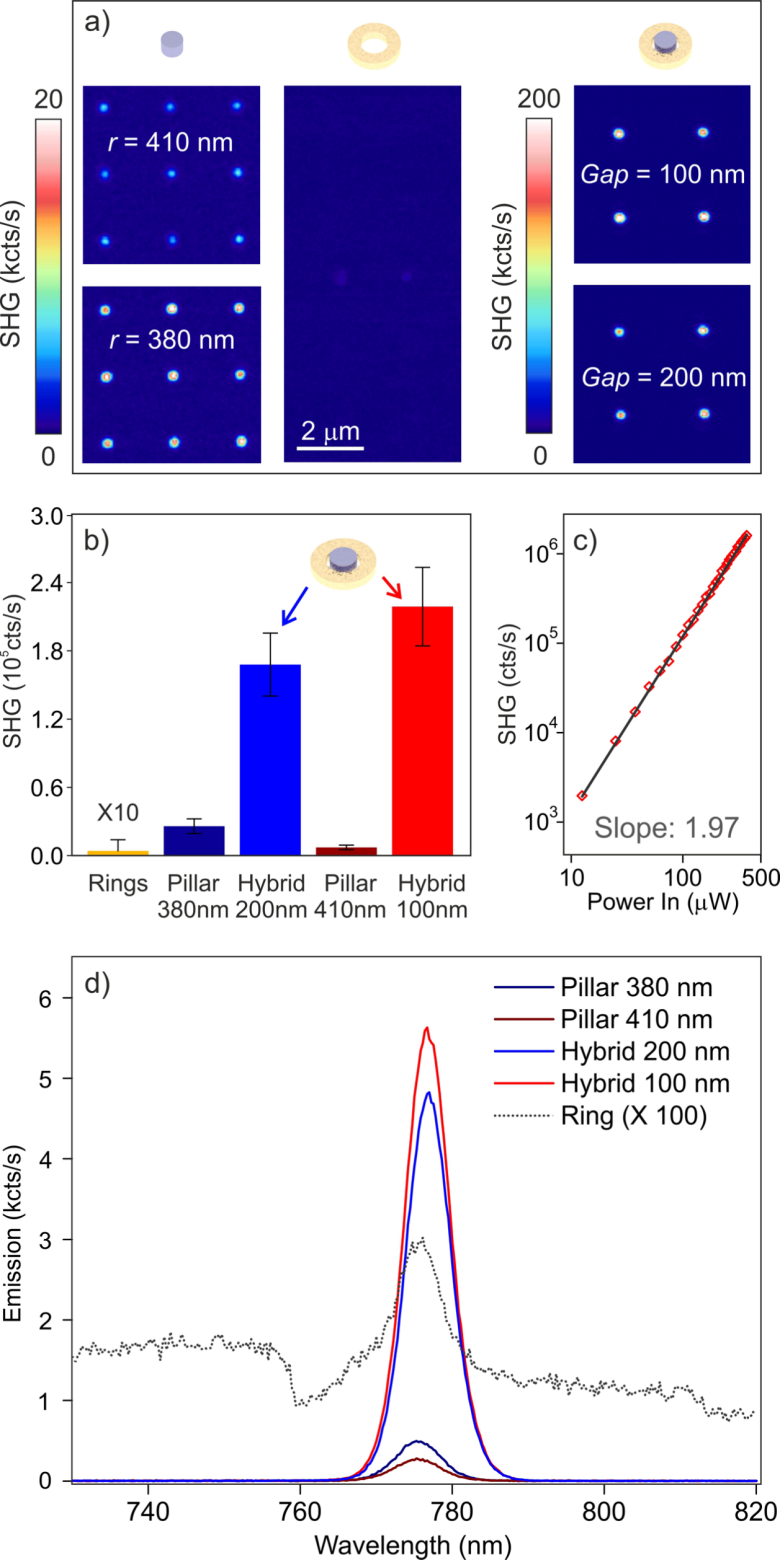
(a) SH nonlinear maps collected from the hybrid antennas and from the reference structures. From left to right: isolated pillars (3 × 3 sub-array), rings (2 × 4 sub array) and hybrid structures (2 × 2 sub-array). (b) Histogram showing the emission statistics for each array. (c) Log–log plot of the emission as a function of the power of structures with a gap of 200 nm, superimposed with a line of slope = 1.97. (d) Nonlinear emission spectra from the different elements under investigation obtained using the same incident pump intensity of 2 GW·cm^−2^.

In this experiment we fully characterized the nonlinear emission from individual nanostructures, by using the nonlinear confocal setup described in the Experimental section (see also [28,33,38]). In brief, each individual nanoantenna is addressed with ultrashort pump pulses (140 fs) centered at 1554 nm and focused down to the diffraction limit by a 0.85 NA objective. The pump peak intensity is kept below a few gigawatts per square centimeter. We measured 10 × 10 arrays of individual pillars, individual Au rings, and the hybrid structures, where the former two are used as references. Figure 4a shows the SHG maps recorded on portions of the arrays: from left to right are shown pillar, rings and hybrid antennas. A statistic of the emission within each array leads to the histogram shown in Figure 4b.

The type-1 configuration (*g* = 100 nm) yields a SHG enhancement factor up to 30 with respect to the corresponding individual pillar (*r* = 410 nm), while for the type-2 configuration (*g* = 200 nm) the signal enhancement is about one order of magnitude. The contribution of the Au ring to the overall SH emission is negligible (Figure 4b), and thus the signal enhancement is only the result of the better field confinement inside the dielectric material brought about by the hybrid configuration, as shown in Figure 3d. A log–log plot of the emission as a function of the power acquired from a type-2 platform using a narrowband filter at 775 nm (25 nm bandwidth) shows the quadratic behavior typical of two-photon processes (Figure 4c). Figure 4d shows the emission spectra collected around the SHG line from the different structures under study. It can be clearly seen that the emission within our window of interest is pure SHG and, along with Figure 3b, this set of measurements quantifies the nonlinear emission enhancement effect due to the hybrid-antenna configuration. Thus, compared to an all-dielectric structure with the same geometrical parameters, the hybrid configuration offers a sizeable nonlinear signal enhancement when the anapole is excited at the fundamental wavelength. To better address this outcome, we have thoroughly analyzed the SHG dependence as a function of the incident polarization as well as the SHG emission patterns for both the hybrid structure and the reference isolated pillar.

**Figure 5:**
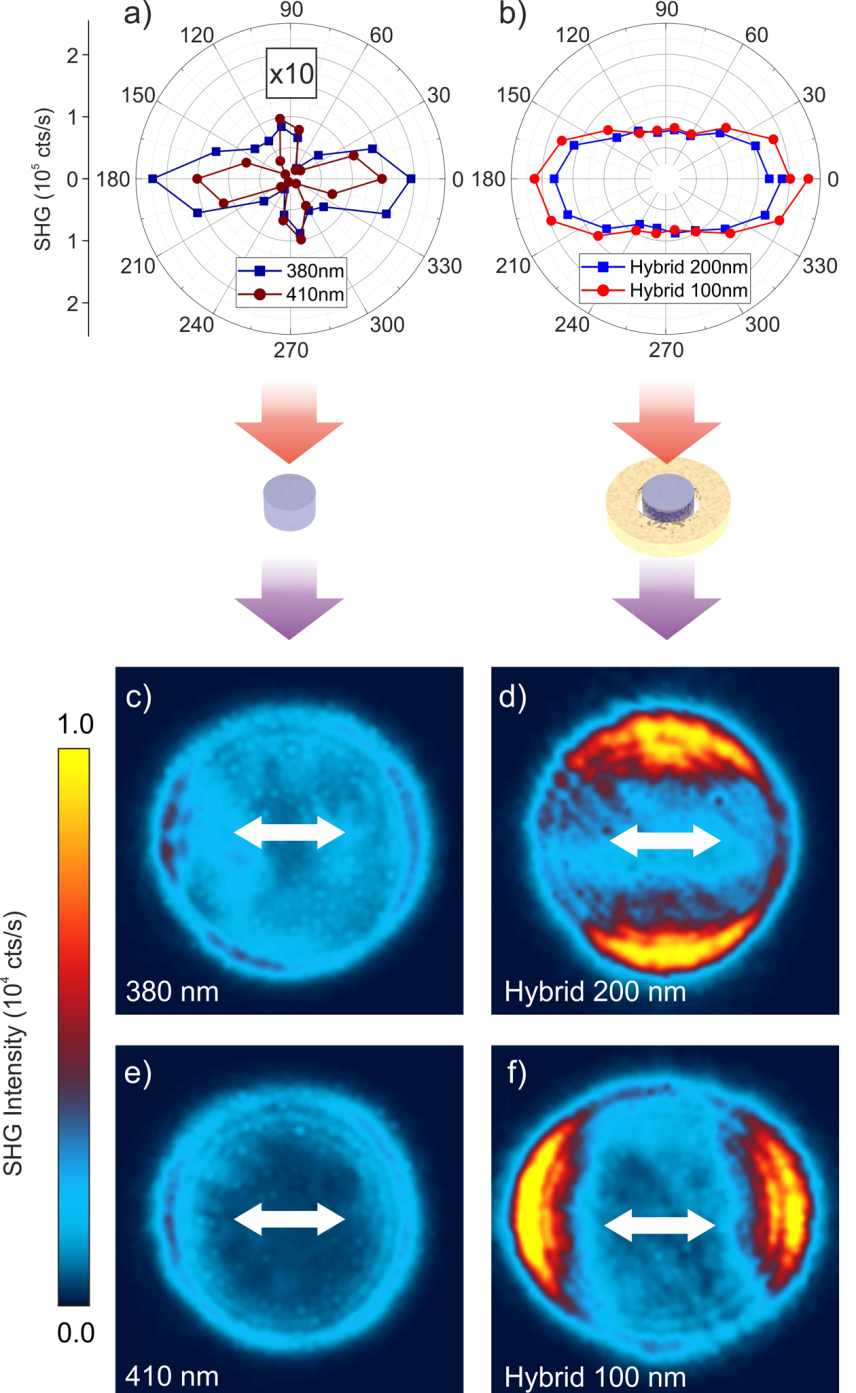
Polar plots of SHG emission from (a) the reference elements and (b) the hybrid antennas as a function of the incident polarization. Experimental emission pattern of the type-1 (c) and type-2 (e) isolated nanopillars used as reference and of their respective hybrid antennas (d) and (f). The white arrow in (c) indicates the polarization direction of the pump field for all the back focal plane maps.

Figure 5 shows the polar plots of the total collected SH intensity (i.e., without an analyzer in the collection path) as a function of the incident light polarization for both the reference elements (Figure 5a) and for the hybrid antennas (Figure 5b). This confirms that the gold ring improves the coupling of the pump light into the crystal by inducing an electric dipole inside the semiconductor that can be oriented parallel to the crystallographic axes for improved SHG. There is also an intensity difference of one order of magnitude between the two sets of polar plots, in agreement with the SHG enhancement factors extrapolated from Figure 4. The emission pattern recorded at the back focal plane for the reference elements (Figure 5c,e) and for the hybrid antennas (Figure 5d,f) confirm an improved collection efficiency for the hybrid configuration. Interestingly, the emission pattern is also strongly modified, moving from an isotropic emission in the reference structures to a dipole-like strongly directional emission in the presence of the plasmonic antenna element.

The highest conversion efficiency achieved by the type-2 hybrid structures is about 5 × 10^−6^, at a pump intensity of about 1.6 GW·cm^−2^. This value is comparable to that of nanopillars of the same material excited at correspondence with the magnetic dipole resonance at the fundamental wavelength, as measured in our previous works [38,41]. This experimental outcome, which is confirmed by our simulations, is the result of a trade-off situation. In fact, while the magnetic dipole resonance in the isolated dielectric nanostructures reported in our early work corresponds to a strong scattering regime, the anapole mode is weakly coupled to radiating modes, therefore it cannot be efficiently excited by far-field illumination. This results in a low field enhancement averaged inside the structure, *F* (blue line in Figure 3d) and in a much higher SHG efficiency for the former platform compared to the nanodisk operating at the anapole condition. In this frame, opening a coupling channel for the light via the gold nanoring helps improving the field enhancement *F* inside the nanodisk, but it also weakens the anapole condition and reduces the quality factor because of increased radiation losses. As a result, while the *F* value in the hybrid antenna is enhanced by more than two orders of magnitude, the SHG yield is only increased by one order of magnitude (see Figure 5d,f). Furthermore, in the hybrid configuration, SHG still gets irradiated at high angles with respect to the antenna axis, hence the overall collection efficiency it not improved substantially. The development of optimized plasmonic nanoantennas for better light coupling and nonlinear emission directionality would allow one to further enhance this process.

**Figure 6:**
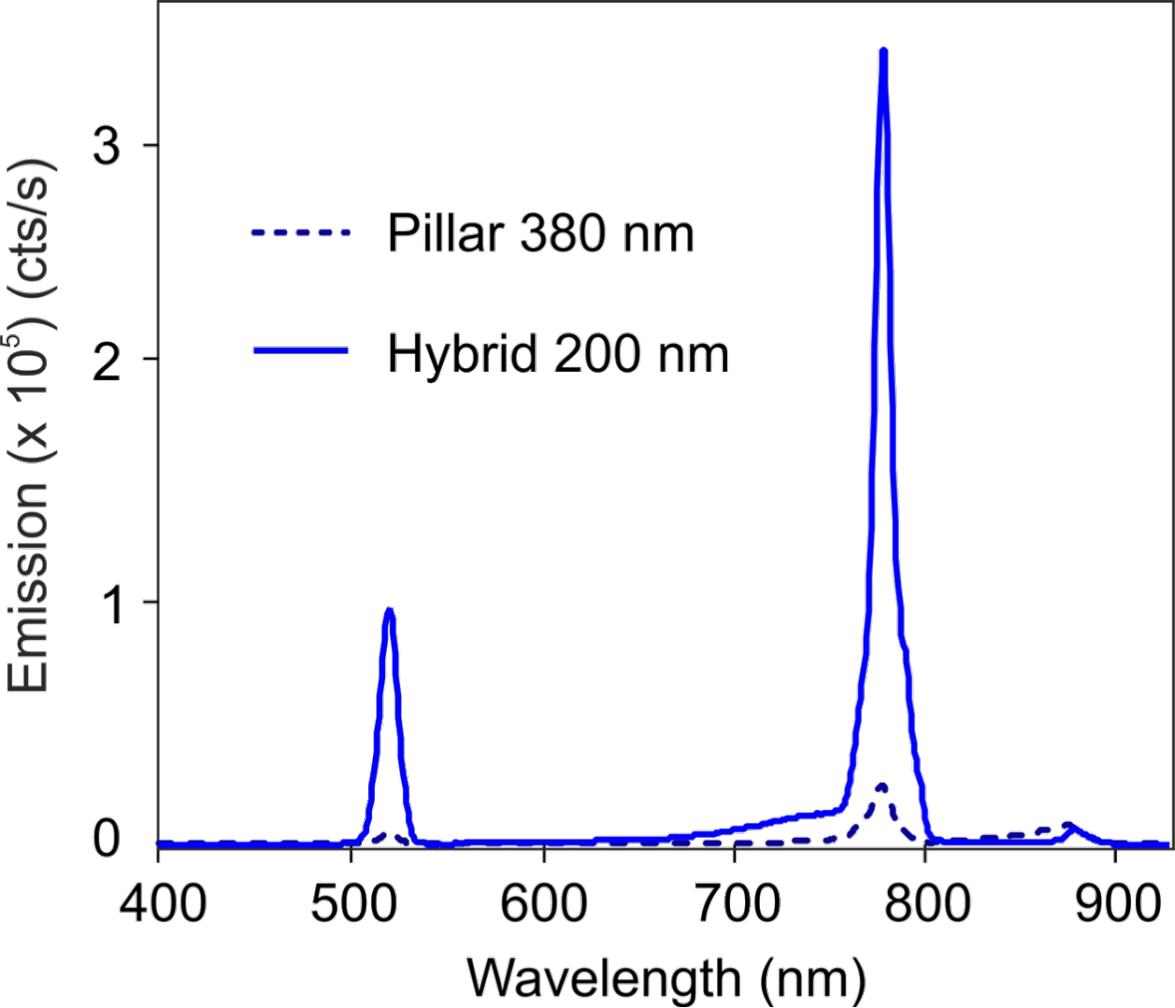
Emission spectrum of a type-2 pillar and its relative hybrid structure. The hybrid structure features more than one order of magnitude enhancement for the THG.

A noteworthy feature of these hybrid nanoantennas, featuring a strong anapole mode-matching at the fundamental wavelength, is also the strong emission yield obtained in THG, which is unexpectedly high for this material, as confirmed by previous investigations [28]. Figure 6 shows the spectra collected from an individual type-2 pillar and its respective hybrid structure, acquired in the whole visible range. While a sizeable THG is already emitted by the reference pillar, the improved field confinement produced by the Au ring in the structure allows one to achieve more than one order of magnitude enhancement in the THG. This corresponds to emission yields comparable with SHG, when an average incident excitation power of about 1 mW is employed.

## Conclusion

We thoroughly investigated a hybrid nanoantenna composed of an individual AlGaAs nanopillar, featuring an anapole mode resonant with the pump wavelength, surrounded by an Au ring designed to improve light coupling to the nanostructure anapole. This enables the improvement of both the second- and third-order nonlinear efficiencies, with measured enhancement factors of about 30 and 15 for the SHG and THG processes, respectively. The analysis of both SHG emission as a function of the pump polarization and angular emission of the SHG reveal that the plasmonic ring increases the pump in-coupling as well as the emission out-coupling. These results represent a step forward in the optimization of nonlinear light manipulation in dielectric nanostructures. The possibility to effectively enhance SHG using the anapole mode field distribution boosted by a plasmonic nanoantenna opens up new avenues for nonlinear sensing, spectroscopy as well as frequency down-conversion and nonlinear multiplexing at the nanoscale [8].

## Experimental

### Sample fabrication

The samples are grown by molecular beam epitaxy from a (100) non-intentionally doped GaAs substrate. At the end of the process, 400 nm of Al_0.18_Ga_0.82_As rest on a 1 μm Al_0.98_Ga_0.02_As sacrificial layer, which will be selectively oxidized at a later stage to achieve a low-index AlO*_x_* substrate. 3 nm of SiO*_x_* are deposited on the surface via plasma-enhanced chemical vapor deposition (PECVD) to later improve the adhesion of the negative-tone HSQ resist on the III–V layer. A first positive-tone lithography step with PMMA resist is performed to realize the alignment structures, followed by electron beam evaporation of 5 nm of Ti and 80 nm of Au. Finally, a standard lift-off procedure reveals the gold alignment structures. The choice of gold as a material at this stage is crucial to ensure the highest possible contrast and precision in the successive lithography step. HSQ resist is then spun and nanoantenna patterns are transferred on the resist with a second lithography step. Two non-selective etching processes are then performed: a first CHF_3_-mediated reactive ion etching (RIE) removes the 3 nm SiO*_x_* layer which is no longer needed, while the second inductively coupled plasma RIE with SiCl_4_/Ar gas treatment transfers the nanoantenna pattern on the Al_0.18_Ga_0.82_As layer, revealing the Al-rich layer. The latter is immediately selectively oxidized at 390° for 30 min under a controlled water vapor flow with N_2_H_2_ carrier gas. Finally, a third positive-tone lithography process analogous to the first one, followed by electron beam evaporation and lift-off, defines the gold nanorings.

### Optical setup

**Figure 7:**
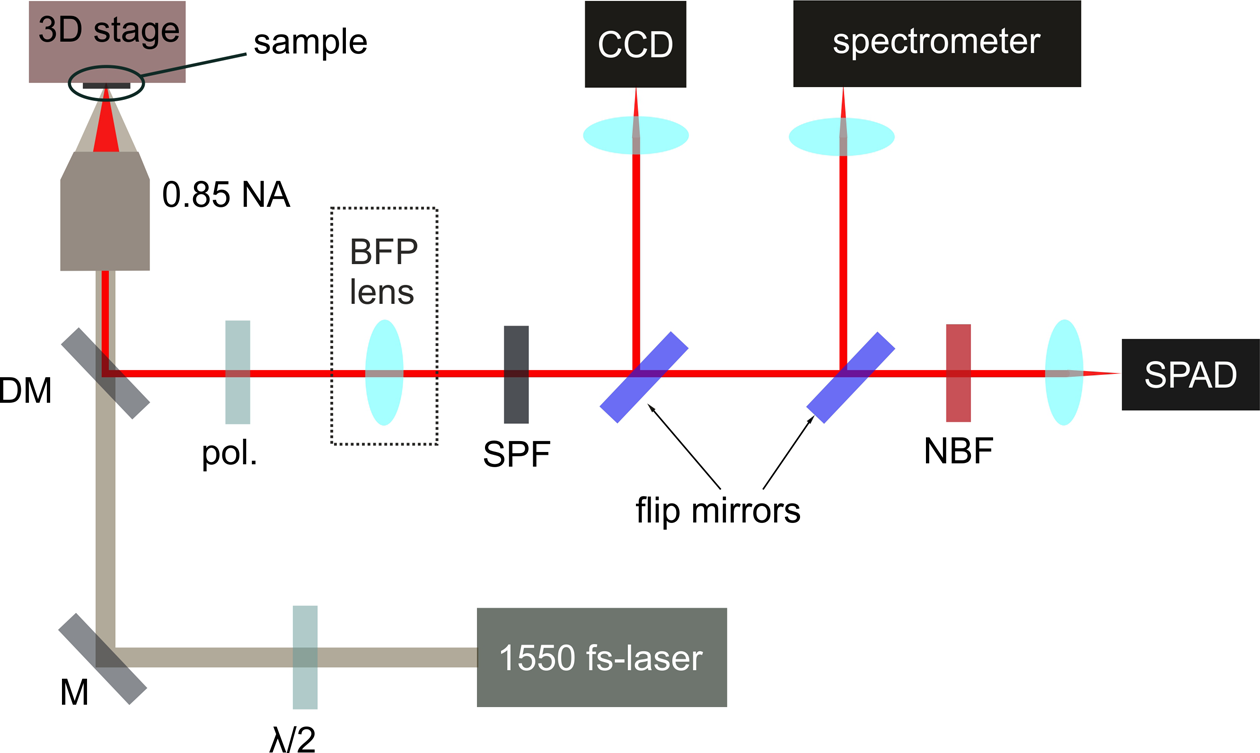
Sketch of the nonlinear setup employed for our measurements. The setup allows for the acquisition of nonlinear maps, nonlinear spectra and nonlinear back-focal-plane images of the pillars. DM, dichroic mirror; BFP lens, back focal plane lens; SPF, short-pass filter; NBF, narrow-band filter; SPAD, single photon avalanche detector.

The nonlinear optical microscope employed in our experiment is depicted in Figure 7. The radiation from a 150 fs-laser centered at 1554 nm is sent to the sample through a high-NA (0.85) air objective, after setting the linear polarization direction with a half-wavelength plate. The nonlinear emission from the antennas is collected in reflection geometry and filtered before detection with a short-pass filter at 1000 nm and with a narrow-band filter at 775 nm to eliminate the residual pump signal and any TH and photoluminescence components. SH maps of the antennas are obtained by raster scanning the sample with a piezoelectric stage, and by recording the signal with a single-photon detector. Once a desired structure has been selected, instead, it is possible to image its SH radiation pattern in the Fourier space on a cooled-CCD camera by inserting a confocal lens focusing at the objective BFP. A flip-mirror can also be inserted in the detection path to send the unfiltered emission to a visible spectrometer for spectrum acquisition.
